# The Effect of 6-Week Combined Balance and Plyometric Training on Dynamic Balance and Quickness Performance of Elite Badminton Players

**DOI:** 10.3390/ijerph19031605

**Published:** 2022-01-30

**Authors:** Zepeng Lu, Limingfei Zhou, Wangcheng Gong, Samuel Chuang, Shixian Wang, Zhenxiang Guo, Dapeng Bao, Luyu Zhang, Junhong Zhou

**Affiliations:** 1 China Institute of Sport and Health Science, Beijing Sport University, Beijing 100084, China; luzepeng@bsu.edu.cn (Z.L.); guozhenxiang@nuaa.edu.cn (Z.G.); 2 School of Strength and Conditioning Training, Beijing Sport University, Beijing 100084, China; zlmf@bsu.edu.cn (L.Z.); luyu_zhang@bsu.edu.cn (L.Z.); 3 School of Physical Education, Jiujiang University, Jiujiang 332005, China; 6030107@jju.edu.cn; 4 Human Biology Major, University of California San Diego, San Diego, CA 92093, USA; smauelchuang2022@gmail.com; 5 Sports Coaching College, Beijing Sport University, Beijing 100084, China; 2020241004@bsu.edu.cn; 6 Department of Physical Education, Nanjing University of Aeronautics and Astronautics, Nanjing 210016, China; 7 Hebrew SeniorLife Hinda and Arthur Marcus Institute for Aging Research, Harvard Medical School, Boston, MA 02131, USA; junhongzhou@hsl.harvard.edu

**Keywords:** balance training, plyometrics training, dynamic balance, quickness, elite badminton player

## Abstract

The study aimed to investigate the effect of combined balance and plyometric training on dynamic balance and quickness performance of elite badminton athletes. Sixteen elite male badminton players volunteered to participate and were randomly assigned to a balance-plyometric group (PB: n = 8) and plyometric group (PT: n = 8). The PB group performed balance combined with plyometric training three times a week over 6 weeks (40 min of plyometrics and 20 min of balance training); while the PT group undertook only plyometric training for the same period (3–4 sets × 8–12 reps for each exercise). Both groups were given the same technical training (badminton techniques for 6 days a week). The dynamic stability and quick movement ability were assessed at baseline and after the intervention by measuring the performance of dynamic posture stability test (*DPSI* and COP), T-running test and hexagon jump test. The results showed that compared to PT, PB induced significantly greater improvements in F-DPSI, L-DPSI (*p* = 0.003, 0.025, respectively), F-COP_AP_, F-COP_ML_, F-COP_PL_, L-COP_PL_ (*p* = 0.024, 0.002, 0.029, 0.043, respectively), T-running test and hexagon jump test (*p* < 0.001). The change in L-DPSI, L-COP_AP_, L-COP_ML_ did not differ between PB and PT (*p* > 0.907). The findings suggest that combined training holds great promise of improving the dynamic balance and quickness performance in elite badminton athletes.

## 1. Introduction

Badminton is one of the fastest racket sports with short intervals and high intensity [1,2]. The badminton players need to make decisions based upon the prediction of the opponent‘s moving direction and the flight trajectory of the badminton in a very short time [3]. This process is closely associated with the capacity of dynamic balance control, including lunges, landing stability and quick adjustment such as acceleration, deceleration, change of direction (COD) of the body trunk [4,5]. The ability to maintain dynamic balance has been linked to increased speed of COD [6], better control of jumping and running to smash, and making the lunges [7]. Therefore, strategies aiming to improve dynamic balance and quickness hold great promise to improve the match performance of badminton players.

One such strategy is plyometric training (PT), consisting of the motions related to muscle eccentric-concentric contraction cycle, also known as the stretch-shortening cycle (SSC) [8,9] (e.g., depth jump and continuous jump [10,11]). PT is widely used in the training of athletes, it can improve jump performance capabilities and strength. Several studies have used it and examined its effects on strength [12], running economy [10], agility [13] and sprint ability [8]. Recent studies have shown that compared to traditional resistance training (RT), PT can induce comparable or even better effects on enhancing the performance of athletes [14,15,16,17] by improving their balance, power, agility and strength. For example, Alikhani et al. [18] observed that a six-week PT could significantly improve dynamic balance and knee proprioception in female badminton players, which can ultimately prevent the injury of participants. Bozdogan et al. [19] also presented that agility and jumping abilities in male badminton athletes were significantly improved after eight-week PT.

More recently, the combined intervention has been developed by implementing two types of training programs at the same time. The combined training can simultaneously improve multiple underlying domains contributing to dynamic balance and quickness, thus inducing greater benefits for the performance compared to the intervention using only one type of training. Studies demonstrated that combined training can significantly improve the strength, balance, and COD ability of basketball and football players [20,21] compared to the intervention using only one type of training. Guo et al., for example, implemented the combined training of balance and PT (PB) in badminton players. This PB training consisted of depth jump and continuous jump with balance exercises on unstable platforms. The results of that study showed that compared to PT only, PB can significantly improve COD performance [22]. However, the effects of this type of combined training of balance and PT on the dynamic balance and quickness in badminton players are not clear.

We here thus in a pilot randomized controlled study aimed to examine the effects of a 6-week combined training on dynamic balance and quickness performance in elite badminton players. Our hypothesis is that a combined training protocol would induce a greater increase in parameters pertaining to dynamic balance and quickness compared to plyometric training.

## 2. Materials and Methods

### 2.1. Participants

Sixteen badminton players were recruited (Table 1). The inclusion criteria were: (1) Elite players who had played the quarterfinalists of national youth games, the finals at the provincial games, or higher-level games; (2) The dominant arm or leg is right side; (3) the ability to complete dynamic balance test and plyometric training; and (4) willing to complete the six-week intervention and the tests. The exclusion criteria were: Participants had ACL, hamstring, meniscus, ankle, or any other lower-extremity injuries that are associated with diminished dynamic balance during the last three years. The study protocol was approved by the Research Ethics Committee of Beijing Sport University (Approval number: 2020008H), and all procedures were conducted in accordance with the Declaration of Helsinki. Before the experiment, participants were informed of the benefits and potential risks related to the study, and all signed the informed consent form.

### 2.2. Study Protocol

Sixteen players volunteered for random allocation to combined balance and plyometric training group (PB, n = 8) or a plyometric group (PT, n = 8). All participants from the PB and PT groups conducted the training program three times per week with 24–48 h of recovery between each training session. The PB group practiced a balance training program (Table S1) on the unstable conditions, and the PT group practiced on the floor with the same training plan. Then PB group and PT group conducted the same plyometric training program (Table S2). Before the formal training and test, all participants completed a 2-week practice (3 times/week) to be familiar with balance exercises, plyometric exercises and the procedure of the test. During this period, participants received proper skill instructions from a strength and conditioning coach. All these training programs have been validated and used to help dynamic balance and quickness performance in athletes. In our previous study, we used the same training protocol in badminton athletes [22]. All the protocols were supervised by the study personnel with experience in strength and conditioning training.

### 2.3. The Assessment of Dynamic Balance and Quickness

The dynamic balance and quickness were assessed at baseline and within three days after the last session of the 6-week training. The tests consisted of Dynamic Posture Stability Test, Modified *t*-test, and Hexagon test. All tests were completed within a day. Before each testing session, participants finished warm-up, including 5-min dynamic stretch, 8-min movement integration, and 2-min neural activation. A 5–10-min rest was given between each test. Each type of test was conducted at the same time and place across different visits, and the participants were asked to wear the same sporting shoes they preferred through all the assessments. The players maintained their normal routine of diet and were prohibited from consuming beverages containing caffeine or alcohol during the whole period. The detailed assessments were as follow:

#### Dynamic Posture Stability Test

The test was used to assess the dynamic balance of players by dynamic posture stability index and center of pressure [23]. Participants stood on an in-ground force plate (Kistler 9281CA, KISTLER, Winterthur, Switzerland, 1000 Hz) and then jumped front or laterad with a dominant leg standing for 10 s. The distance between the jumping line and the center of the force plate was 40% of the players’ height (cm) [23,24]. The fence was placed at the midpoint of the connection between those. The fence height in the jumping front was 30 cm, and that in jumping laterad was 15 cm. All players were asked to complete two kinds of jumps three times, and the average would be taken for data analysis. Matlab software (r2014b, MathWorks, Natick, Massachusetts, USA) was used to calculate dynamic postural stability index (*DPSI*) and center of pressure (COP). The time-series data of ground reaction force (GRF) and center of pressure (COP) were collected within 10 s after players landed on the force plate with a single leg. All data were smoothed through low-pass filtering, and the truncation frequency was set to 13.33 Hz.

*DPSI* was calculated from the GRF curve within 3 s after touchdown (the time when the GRF value exceeded 5% of body weight) [25]. Where BW is the body weight, GRF_x_, GRF_y_, and GRF_z_ are the back and forth, left and right, vertical ground reaction forces. The dynamic posture stability indexes of forward jump (DF-DPSI) and lateral jump (DL-DPSI).

(1) $$DPSI=\frac{\left(\frac{\sqrt{\sum {\left(0-{\mathrm{GRF}}_{x}\right)}^{2}+\sum {\left(0-{\mathrm{GRF}}_{y}\right)}^{2}+\sum {\left(\mathrm{BW}-{\mathrm{GRF}}_{z}\right)}^{2}}}{\;\mathrm{number}\;\mathrm{of}\;\mathrm{data}\;\mathrm{points}\;}\right)}{\mathrm{BW}}$$

COP was calculated from the time series within 10 s after landing, and the back and forth displacement difference (COP_AP_), left-right displacement difference (COP_ML_) and total displacement distance (COP_PL_) of forwarding jump (DF) and lateral jump (DL) were calculated [14]. x_t_ and y_t_ are the back and forth, left and right displacements at t seconds and the value of t is 1–10 s.

(2) $${\mathrm{COP}}_{\mathrm{AP}}=\sum_{1}^{10}{\left(x_{t}\;-\;\bar{x}\right)}^{2}$$

(3) $${\mathrm{COP}}_{\mathrm{ML}}=\sum_{1}^{10}{\left(y_{t}-\;\bar{y}\right)}^{2}$$

(4) $${\mathrm{COP}}_{\mathrm{PL}}=\sum_{0}^{9}\sqrt{{\left(x_{t+1}-x_{t}\right)}^{2}+{\left(y_{t+1}-y_{t}\right)}^{2}}$$

### 2.4. Modified t-Test

*t*-test was widely used for evaluating the quickness of athletes in various sports [26]. To fit the feature of badminton specifically, researchers adjusted the distance to be similar to the badminton court, as shown in Figure S1. Cone bucket A was placed at the starting line, and then cone bucket B was set at 6 m away from A. Cone buckets C and D were placed at 3 m to B side to side. Smart Speed device (Fusion Sport, Coopers Plains, Australia) was set on A. After hearing the order “Ready, go”, the players started to forward sprint from A to B with left-hand touching bucket B. Then they made side shuffling from B to C with left-hand touching bucket C. Upon arrival, they did the same side shuffling to D with right-hand touching and made the same shuffling to B. Finally, the players did backpedal to A. Each participant had to perform three times, and the best of results was the final valid score.

### 2.5. Hexagon Test

The test was applied to assess COD ability for badminton and was verified as an effective method to evaluate the on-court performance of players [27,28]. The participants stood 50 cm behind the No. 1 side of the hexagon (Figure S2), and after hearing the command of “Ready, go”, they quickly completed jumping in and out of the line with a circle clockwise from 1 to 6. Smart speed devices would be timed automatically at the beginning and end of the test, with a total of three times, of which the shortest time was recorded for analysis. There was a 2-min passive rest between the two tests.

### 2.6. Statistical Analysis

Experimental data were processed by IBM SPSS statistical software package (version 25.0, IBM, Chicago, IL, USA). All data were presented as means and SD.

To examine the effects of the combined training on the performance of dynamic balance ability, *t*-test and Hexagon test, we performed two-way repeated-measure ANOVA (group × time), and the Greenhouse-Geisser adjustment was applied. The dependent variable for each model was *DPSI* (front and lateral), COP (AP, ML, PL), the time of *t*-test and Hexagon test. The model effects were group, time and their interaction. When a significant model effect was observed, LSD post-hoc correction was performed to identify significant pairwise differences. Partial η^2^ was used to assess the effects size where the significance was observed. The level of significance was set at *p* < 0.05 for all tests.

## 3. Results

### 3.1. Dynamic Balance Ability

#### 3.1.1. Dynamic Posture Stability Index

The results of ANOVA models on F-DPSI and L-DPSI showed the significant effects of time (F-DPSI: *p* < 0.001; L-DPSI: *p* < 0.001), that is, compared to baseline, participants in both groups had significantly lower F-DPSI and L-DPSI after the intervention. A significant interaction between group and time (F-DPSI: *p* = 0.003; L-DPSI: *p* = 0.025) was also observed (Table 2). The post-hoc analysis revealed that the participants who received PB had significantly lower F-DPSI and L-DPSI as compared to their baseline performance and those who received PT. [F_(1,28)_ = 29.661, *p* < 0.001, partial η^2^ = 0.514]. No significant effects of group were observed (F-DPSI: *p* = 0.418; D-DPSI: *p* = 0.593).

#### 3.1.2. Center of Pressure

Significant effects of time and group, but non-significant interaction between group and time on F-COP_AP_ [time: *p* = 0.008; group: *p* = 0.024], F-COP_ML_ [time: *p* = 0.008 respectively; group: *p* = 0.002], F-COP_PL_ [time: *p* = 0.036; group: *p* = 0.029] and L-COP_PL_ [time: *p* = 0.01; group: *p* = 0.043] were observed. Specifically, compared to baseline, the F-COP_AP_, F-COP_ML_, F-COP_PL_, and L-COP_PL_ were significantly lower after the intervention, and these outcomes in PT group were significantly greater than those in PB group. Additionally, significant effects of time (L-COP_AP_: *p* = 0.005; L-COP_ML_: *p* = 0.023), but no significant interaction between group and time (L-COP_AP_: *p* = 0.283; L-COP_ML_: *p* = 0.907) on L-COP_AP_ and L-COP_ML_ were observed.

### 3.2. Quickness Performance

Significant effects of time (*t* test: *p* < 0.001; hexagon test: *p* < 0.001) were observed on the performance of *t* test and hexagon test, while only trends toward significance were presented for the effects of group (*t* test: *p* = 0.106; hexagon test: *p* = 0.052) and the interaction between group and time (*t* test: *p* < 0.001; hexagon test: *p* < 0.001). Specifically, the post-hoc analysis revealed that for both PB and PT group, *t*-test and hexagon test were significantly greater than baseline; and participants who received PB tend to have significant lower *t*-test and hexagon as compared to their baseline performance and those who received PT [*t*-test: F_(1,28)_ = 5.139, *p* = 0.031, partial η^2^ = 0.155; hexagon test: F_(1,28)_ = 4.811, *p* = 0.037, partial η^2^ = 0.147].

## 4. Discussion

The results of this pilot study demonstrated that dynamic balance and quickness performance in elite badminton athletes were significantly improved after combined training as compared to those who received PT only, suggesting combined training is thus a promising strategy to improve the functionality of elite badminton athletes and potentially help their on-court performance.

It is observed that compared to the intervention using plyometric training only, 6-week combined training of balance and plyometric training can significantly improve the ability to control dynamic balance and quickness performance in elite professional athletes of badminton as assessed by using the task of dynamic balance ability, *t*-test, and Hexagon test. The performance of *DPSI* and COP reflects the ability of players to maintain landing stability and balance [25,29]. The traditional PT oftentimes focuses only on the musculoskeletal function, while the combined training can simultaneously target multiple aspects pertaining to dynamic balance, such as the integration of the visual, vestibular information and the coordination across those systems [30]. This can thus benefit the capacity of postural control system to appropriately reweight and utilize different types of sensory inputs (e.g., visual, proprioceptive) when receiving the challenges or perturbations. For example, Nepocatych et al. [31] observed that balance training had the potential to induce adaptive responses in the neuromuscular system that enhances postural control, the balance of women, as well as improvement of COP to prevent chronic ankle instability effectively. Additionally, previous studies demonstrated that balance training would lead to spinal reflex during postural movement, which results in less destabilizing movement [32]. Therefore, the combined training is a strategy that would be more appropriate for dynamic balance by simultaneously enhancing multiple underlying elements of dynamic balance control.

Quickness is another important factor for outstanding performance in badminton competitions [33]. Quickness not only requires the power of lower limbs to change body direction but also has good dynamic balance ability to control body posture so as to overcome the inertial effect caused by acceleration and deceleration braking in the process of changing direction [24]. The rounds of multiple rallies occur more frequently in the badminton match [34]. Players thus need to judge the direction and trajectory of the ball quickly before hitting the ball, then quickly change the direction of body, and perform acceleration and deceleration of the movement [2]. Previous studies have shown that combined training improves the performance of change of direction in college-level badminton players [22], as well as in other participant cohorts [20,35,36]. For example, eight weeks of combined training induces significantly greater improvements in the performance of Illinois change of direction in woman basketball players compared to PT intervention [37]. Here we further demonstrate that the combined training can induce significantly greater benefits for quickness in elite badminton players compared to traditional PT. Specifically, the balance training can enhance the adaptive capacity for the rate of force development [38], and increase muscle activation during the landing of jump procedure [39], which might optimize musculotendinous and joint stiffness. The musculotendinous and joint stiffness are critical to the smoothness of action, one critical aspect of quickness [40]. Therefore, the combined training could be considered as a training program in increasing both the performance of dynamic balance and quickness. There are some limitations in this study. First, the sample size of 16 people is relatively small as we focused on only elite athletes, future studies with a larger sample size on other cohorts (e.g., older adults) are thus highly demanded. Second, this study implemented a 6-week intervention with only one follow-up assessment. The optimal intervention intensity and the dose-response relationship between the performance and the “dose” of the intervention are still unknown. Future studies with multiple visits during and after the intervention and longer-term follow-up period are thus needed to determine how long the effects of combined training can sustain and the appropriate intensity, the number of sessions of the combined training that can maximize the benefits of the combined training. This will ultimately help the optimized design of the PB intervention. Additionally, we only included one control group that received PT training. It is worthwhile to include a blank control group so that the elevated benefits from the control group to single-type training to PB can be examined. Future studies implementing more sophisticated measurements of the characteristics of the underlying musculoskeletal system and sensory perception are needed to explore the mechanism through which combined training can induce greater benefits for dynamic balance and quickness compared to single-type training.

## 5. Conclusions

This pilot study showed that balance training combined with plyometric training can strengthen dynamic balance ability and improve the quickness performance of male elite badminton players. The knowledge obtained from this study will ultimately help inform the design of future larger-scale studies to confirm the findings in this study.

## Figures and Tables

**Table 1:** The demographic characteristics of the participants.

	Age (Years)	Height (cm)	Weight (kg)	Training Experience (Years)
PB (n = 8)	20.50 ± 1.07	177.75 ± 5.06	68.13 ± 7.22	11.38 ± 1.41
PT (n = 8)	19.13 ± 2.23	179.13 ± 6.06	69.88 ± 8.94	10.63 ± 1.06

No significant difference in the demographic characteristics between PB group and PT group (*p* > 0.066).

**Table 2:** The assessment results for PB group and PT group before and after the 6-week training.

		PB (N = 8)			PT (N = 8)		
		Pre	Post	Partial η^2^	Pre	Post	Partial η^2^
Dynamic balance	F-DPSI	0.386 ± 0.002	0.381 ± 0.003 *^#^	0.653	0.387 ± 0.002	0.384 ± 0.001 *	0.207
	L-DPSI	0.386 ± 0.003	0.378 ± 0.002 *	0.543	0.384 ± 0.004	0.381 ± 0.003 *	0.175
	F-COP_AP_ (cm)	90.28 ± 16.39	72.20 ± 10.81 *^#^	0.233	95.15 ± 12.65	88.27 ± 8.28	0.042
	F-COP_ML_ (cm)	72.97 ± 11.99	60.55 ± 6.23 *^#^	0.192	81.90 ± 10.30	74.81 ± 9.08	0.072
	F-COP_PL_ (cm)	131.60 ± 22.10	109.70 ± 18.56 *^#^	0.187	137.27 ± 15.39	132.19 ± 11.17	0.012
	L-COP_AP_ (cm)	79.29 ± 12.35	63.37 ± 9.83 *^#^	0.236	82.44 ± 10.31	74.90 ± 10.68	0.065
	L-COP_ML_ (cm)	90.71 ± 10.32	81.37 ± 10.18 *	0.102	93.64 ± 11.66	85.17 ± 9.65	0.085
	L-COP_PL_ (cm)	131.25 ± 19.38	110.05 ± 16.89 *	0.176	139.82 ± 14.69	127.44 ± 18.14	0.068
Quickness performance	Hexagon test (s)	3.83 ± 0.32	2.95 ± 0.14 *^#^	0.592	3.95 ± 0.29	3.25 ± 0.33 *	0.472
	*t*-test (s)	7.38 ± 0.19	6.77 ± 0.11 *^#^	0.637	7.39 ± 0.24	6.96 ± 0.13 *	0.455

Note: * Statistically significant difference between pre- and post-test, *p* < 0.05. ^#^ Statistically significant difference between PB group and PT group, *p* < 0.05. F, forward jump; L, lateral jump; COP, center of pressure.

## Supplementary Materials

The following supporting information can be downloaded at: https://www.mdpi.com/article/10.3390/ijerph19031605/s1, Figure S1: Modified *t*-test; Figure S2: Hexagon test; Table S1: The balance training program for balance-plyometric (PB) (combined training) group and Table S2: The plyometric training (PT) program for PB and plyometric training group.
