# Supplementary material for: The Effect of 6-Week Combined Balance and Plyometric Training on Dynamic Balance and Quickness Performance of Elite Badminton Players
Source: Int J Environ Res Public Health. 2022 Jan 30;19(3):1605. doi: 10.3390/ijerph19031605 (PMC8835527; doi:10.3390/ijerph19031605)
Supplement: Supplementary file 1 [file ijerph-19-01605-s001.zip › ijerph-1553104-supplementary.pdf]

Table S1: The balance training program for balance-plyometric (PB) (combined training) group.

Exercises	The first stage (1-2 weeks)	The second stage (3-4 weeks)	The third stage (5-6 weeks)
Stand on the balance board exercise	Static standing on the board with two legs (3 sets: 30 s/set)	Static standing on the board with two legs and eyes closed (3 sets: 30 s/set)	Squat on the plate with eyes closed (3 sets: 10 reps/set)
Supine straight leg bridge on Swiss Ball	Isometric supine straight leg bridge on Swiss Ball (3 sets: 30 s/set)	Isometric supine single-leg bending bridge on Swiss Ball (3 sets: 30 s/set)	Dynamic supine single-leg bending bridge on Swiss (3 sets: 10 reps/set)
Side-plank with inflated balance disc	Side-plank with inflated balance disc with elbow (3 sets: 30 s/set)	Side-plank with inflated balance disc and the non-supporting leg stretches backward (3 sets: 10 reps/set)	Side-plank with inflated balance disc and the non-supporting leg stretches backward with elastic band (3 sets: 10 reps/set)
Lunge squat on BOSU ball	Lunge squat on BOSU ball (3 sets: 10 reps/leg/set)	Lunge squat on BOSU ball and inflated balance disc (3 sets: 10 reps/leg/set)	Lunge squat on BOSU ball and inflated balance disc with 5 kg dumbbells (3 sets: 10 reps/leg/set)
Airex® Balance-pad Elite exercise	Single-leg squat with balance-pad (3 sets: 10 reps/leg/set)	Single-leg standing with balance-pad and the non-supporting leg stretches backward (3 sets: 12 reps/leg/sets)	Single-leg support with balance-pad elite and the non-supporting leg stretches backward with elastic band (3 sets: 12 reps/leg/sets)
Rest	Between exercise: 60 s Between sets: 3 min		

Table S2: The plyometric training (PT) program for PB and plyometric training group.

Exercises	The first stage (1-2 weeks)	The second stage (3-4 weeks)	The third stage (5-6 weeks)
Front barrier jump (6 hurdles)	Double-leg front barrier jump (15 cm) (3 sets: 10 reps/set)	Single-leg front barrier jump (15 cm) (3 sets: 5 reps/leg/set)	Single-leg front barrier jump (30 cm) (4 sets: 5 reps/leg/set)
Lateral high-knees with hurdles	4-hurdle (15 cm) (3 sets: 2 reps/set)	6-hurdle (30 cm) (3 sets: 4 reps/set)	6-hurdle (30 cm) (3 sets: 6 reps/set)
Lateral barrier jump	Double-leg jump (15 cm) (3 sets: 10 reps/set)	Double-leg jump (30 cm) (3 sets: 12 reps/set)	Single-leg jump (30 cm) (3 sets: 15 reps/leg/set)
Depth jump	Jump with 20 cm box (3 sets: 8 reps/set)	Jump with 30 cm box (3 sets: 8 reps/set)	Jump with 40 cm box (3 sets: 8 reps/set)
Multi-direction jumps with hurdles	Triangle jump with double-leg (3 hurdles) (3 sets: 6*3 reps/set)	Square jump with single-leg (4 hurdles) (3 sets: 8*3 reps/set)	Hexagon jump with single-leg (6 hurdles) (3 sets: 12*3 reps/set)

Intensity and number of contact with ground	Low intensity 144	Middle intensity 234	High intensity 325
Rest	Between exercise: 60 s Between sets: 3 min		

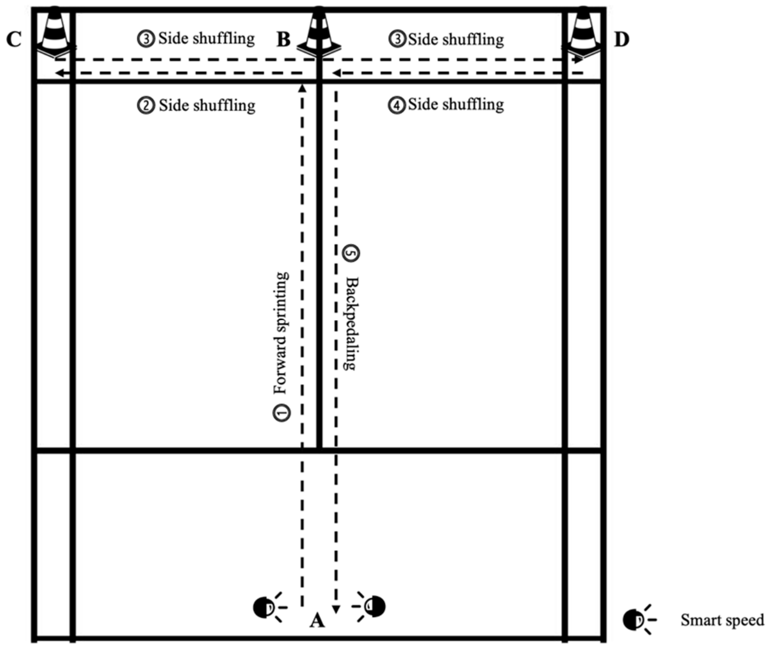

Figure S1. Modified T-test

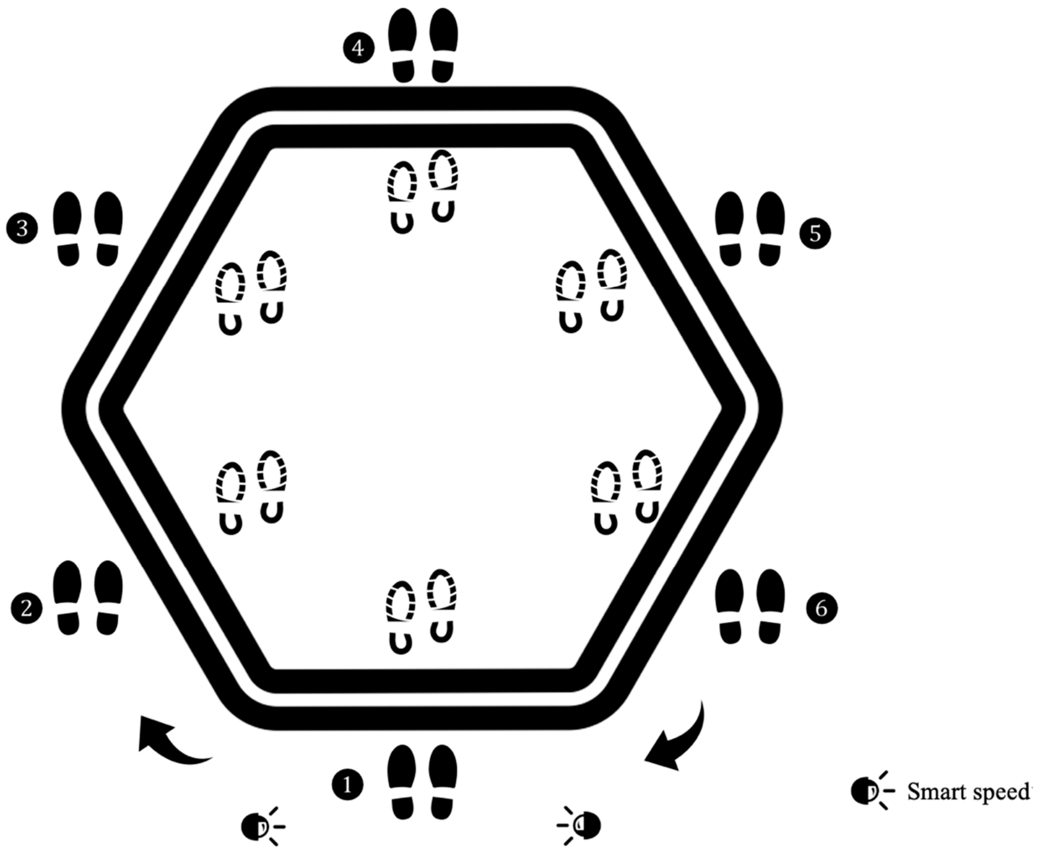

Figure S2. Hexagon test
